# Determination of the role of calcium on instability of neurotoxic metabolite of ecstasy by HPTLC-mass

**DOI:** 10.1186/2008-2231-21-9

**Published:** 2013-01-17

**Authors:** Bardia Jamali, Yalda Hosseinzadeh Ardakani, Mohammad-Reza Rouini, Alireza Foroumadi, Salimeh Amidi, Vahid Hossein Zadeh Aghdam, Farzad Kobarfard

**Affiliations:** 1 Department of Pharmaceutics, Biopharmaceutics and Pharmacokinetics Division, Faculty of Pharmacy, Tehran University of Medical Sciences, Tehran, Iran; 2 Drug design and Development Research Center, Tehran University of Medical Sciences, Tehran, Iran; 3 Department of Medicinal Chemistry, School of Pharmacy, Shahid Beheshti University of Medical Sciences, Tehran, Iran; 4 Central Research Laboratories, Shahid Beheshti University of Medical Sciences, Tehran, Iran

**Keywords:** HHMA, HPTLC-MS, Stability, Calcium chloride, Neurotoxicity

## Abstract

**Background:**

Ecstasy is one of the popular illicit drugs in the world and its usage has been recently increased in Iran. This compound can destroy the serotonergic neurons and produces cognitive and psychopathology diseases. 3,4-dihydroxymethamphetamine (HHMA) which is the main metabolite of this compound, seems to be responsible for this effect. However, no consensus has been reached among the researchers about its role. This disagreement between the researches may be due to failure in determination of HHMA as free form in physiological fluids. In this study, the stability of this crucial metabolite of ecstasy was examined in different mediums.

**Methods:**

The stability of HHMA was studied in the perfusion medium and water at 100 and 10 ng/mL concentrations. Moreover, the effect of temperature (0–25°C), pH (3–10), calcium chloride (0–150 g/L) and ethylenediaminetetraacetic acid (EDTA) on the stability of HHMA was also examined.

**Results:**

Our result suggested that the free form of HHMA could be degraded in the perfusion medium. The rate of this degradation has direct proportion to temperature (at 25°C = 0.037 min^-1^ and at 0°C = 0.002 min^-1^). Calcium chloride and sodium bicarbonate are two responsible components in this instability. Moreover, the alkaline pHs and increasing the shaking time can accelerate this effect. Although, while degradation was prevented at pH=3, EDTA could only reduce this rate about 30%.

**Conclusions:**

Calcium cation can act as an accelerator of HHMA degradation. Therefore, the perfusion medium should not contain Ca^2+^ and the pH of medium is better to be adjusted at acidic range. Since, the internal cellular source of calcium is endoplasmic reticulum system, it can be assumed that, this cation may change HHMA and dopamine to reactive compounds that can bind covalently to the cysteinyl group of biological compounds and damage cellular components.

## Introduction

Ecstasy (3,4-methylenedioxymethamphetamine, MDMA) is one of the popular drugs of abuse among youths. The tendency toward this compound has increased since the last two decades [1,2]. In 2009, it was reported that 2.8 million of Americans over 12 years old have abused MDMA at least once [3]. The usage of this compound has been recently increased among Iranian youths as well [4,5].

MDMA is a releaser and reuptake inhibitor of serotonin and dopamine which can destroy serotonin axon terminal of brain and produces cognitive dysfunction and psychopathology diseases in humans. However, the mechanism behind this phenomenon is not clearly known [2,6,7]. It was reported that the intracranial administration of MDMA in animals does not produce any neurotoxic effects. However, the peripheral administration can do so [7]. This result and some other reports proposed that MDMA metabolites may have a role in its neurotoxic activity [7,8].

**Figure 1:**
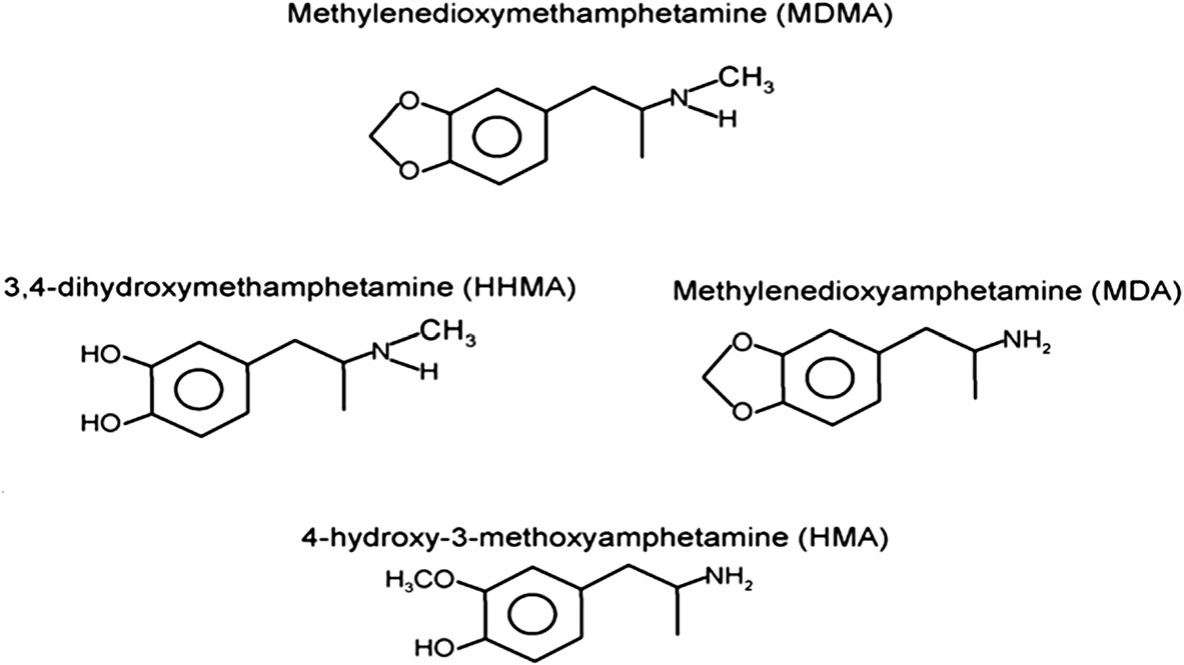
Structure of MDMA and its metabolites

MDMA is metabolized via either O-demethylation or N-dealkylation in humans and rats. N-dealkylation pathways produce 3,4-methylenedioxyamphetamine (MDA) which is known as “Love drug” and has similar pharmacological effects to MDMA [1]. O-demethylation produces reactive catechol metabolite, i.e. 3,4-dihydroxymethamphetamine (HHMA) (Figure 1). HHMA is the main metabolite of MDMA in urine and plasma which exist in conjugated form (sulfate and glutathione) and there is no report for determination of its free form in biological fluids [2,7]. It has been reported that HHMA conjugated with glutathione could produce apoptosis on rat hipocampal neurons [8].

Over the years, different reports have been published about the neurotoxicity associated with MDMA metabolites. Some reports proposed that MDA is responsible for such an effect [6] and the others have introduced HHMA and thioether adducts as the toxic metabolite which cause apoptosis and damage in the cells [7,8]. This controversy may be due to the failure in determination of HHMA as a free form in brain after MDMA administration.

**Figure 2:**
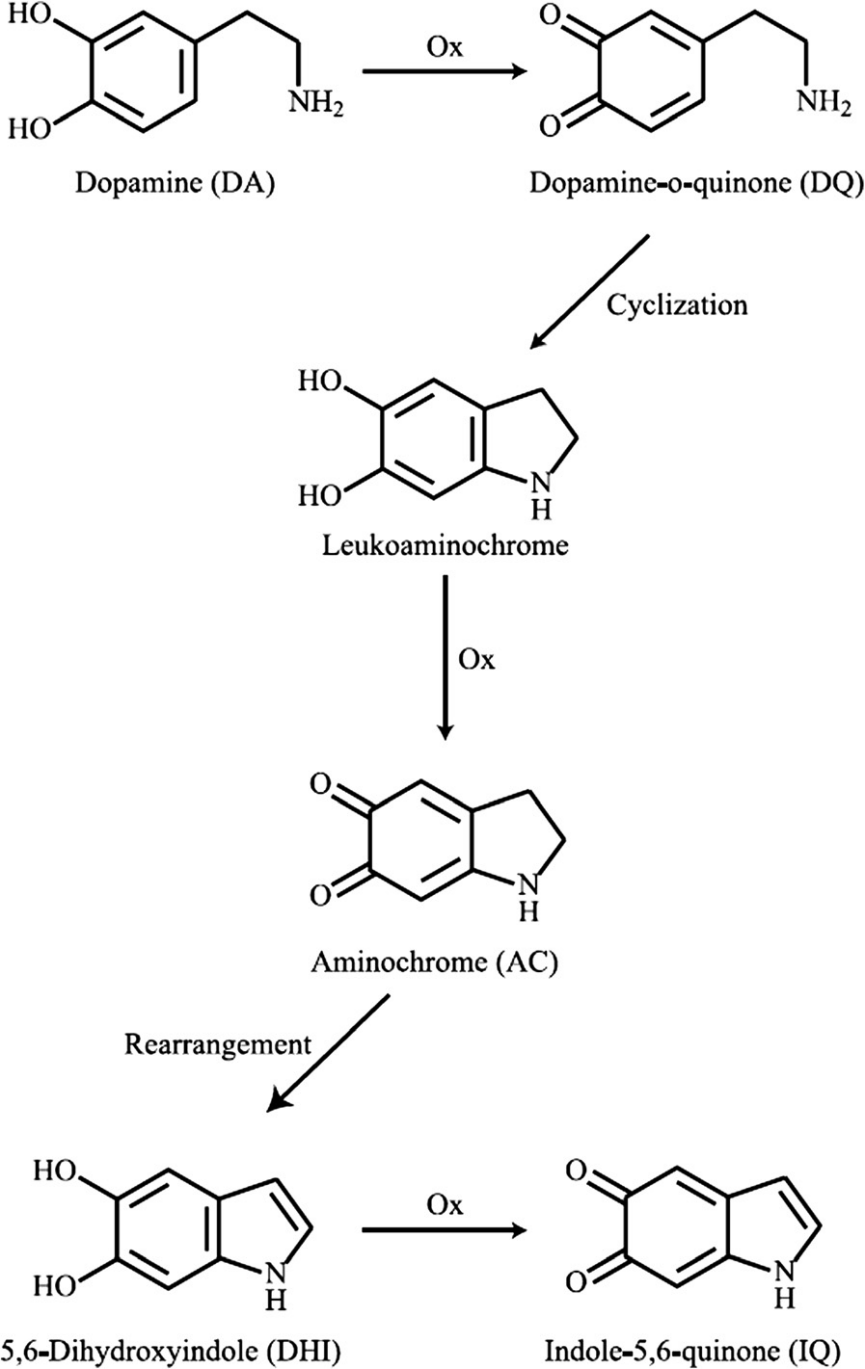
**The oxidation pathway of dopamine**[10]**.**

HHMA, due to its catecholamine structure, can display various properties. The catecholamine moiety is prone to oxidation and formation of ortho quinone-like structure. Oxidizing agents such as ClO^-^, MnO_4_^-^ and etc., alkaline pHs and light can trigger this reaction [9]. Moreover, MDA can also be O-demethylated and form another metabolite with catecholamine structure which can exert similar properties with HHMA [2]. To the best of our knowledge, there are no reports about HHMA stability in physiological fluids and there are only some reports regarding the stability of dopamine which has the closest structure to HHMA. These reports indicate that dopamine, in the presence of oxidizing agents like Mn^3+^ or NaIO_4_, is converted to the reactive compounds such as aminochrome (AC), indole-5,6-quinone and 5,6-dihydroxyindole. These reactive compounds can bind covalently to the cysteinyl group of biological compounds in the body and damage cellular components such as DNA, proteins and lipids. The role of these reactive compounds in Alzheimer’s and Parkinson’s has been established (Figure 2) [10,11].

Due to the ethical and safety issues, studying the metabolism changes of illicit or toxic compounds at high concentration in human subjects is constrained. Therefore, the kinetic of these compounds is preferred to be studied in similar systems to human such as isolated animal organs. In these cases, the perfusion medium is used instead of plasma [12]. However, the stability of the compounds and their related metabolites in the perfusion medium is of important concern. Since MDMA is an illicit compound and can cause multi organ failure, especially liver damage and death at high concentrations [13], its metabolic transformation is preferred to be studied in intact animals or in in vitro perfused organs.

In the present study, stability of HHMA in perfusion medium was examined. The constituents of this medium appear in almost all of physiological fluids of human body. Determination of the possible effect of these constituents on the stability of HHMA free form could help researcher to determine the role of HHMA in MDMA neurotoxicity.

## Materials and methods

### Chemicals

Ultrapure water was obtained from Millipore Direct-Q system (France). HHMA was synthesized in four steps, from commercially available 3,4-dimethoxy-benzaldehyde and nitroethane, through procedures previously reported [8]. Dopamine was a gift from Caspian Tamin Pharmaceutical Company. HPLC (high performance liquid chromatography) grade methanol and all other chemicals of analytical grade were purchased from Merck (Darmstadt, Germany). The Krebs buffer (6.903 g/L NaCl, 0.335 g/L KCl, 0.162 g/L KH_2_PO_4_, 0.163 g/L MgSO_4_, 0.305 g/L CaCl_2_, 2.1 g/L NaHCO_3_ and 1 g/L glucose, pH was adjusted to 7.2-7.4) was used as a perfusion medium.

### Apparatuses and chromatographic conditions

The Knauer chromatographic system which was used, consisted of a pump model K-1001, a fluorescence detector (FL) model RF-10AXL, a photodiode array (PDA) detector model K-2800, a solvent degasser and a 100 μl loop injector. EZChrom Elite software was used for instrument control, data acquisition and analysis. The separation of analytes was performed at ambient temperature (25°C) on the Chromolith® Performance RP-18e (100 × 4.6 mm) column with Chromolith® RP-18e (5 × 4.6 mm) guard from Merck (Darmstadt, Germany). The FL detector wavelength was fixed at 285 nm for excitation and at 320 nm for emission and PDA wavelength was fixed at 285 nm. The mobile phase was 0.02 M potassium dihydrogen phosphate (KH_2_PO_4_) solution with pH = 3 (orthophosphoric acid was used for pH adjustment).

The Cecil UV spectrophotometer model CE9500 and Camag high performance thin layer chromatographic (HPTLC) system consisted of a Camag TLC Scanner 3, a TLC automatic developing chamber and a Camag automatic TLC Sampler 4 with a PDA and FL detectors, were used. In the HPTLC system, the separation was performed on HPTLC plates coated with silicagel 60 F_254_ (10 × 10 cm) from Merck (Darmstadt, Germany). A mixture of dioxane:methanol:chloroform:acid acetic (6:1:1:1,v/v/v/v) was used as the mobile phase. The instrument was controlled and the data were analyzed by winCATS planar chromatography manager software. TLC plates developed by HPTLC were subjected for TLC-Mass spectrometry using a 1200 Agilent HPLC pump coupled to a Agilent electrospray ionization (ESI) mass detector (6410 Triple Quadrupole) through a Camag TLC-MS interface. Methanol was used for elution of TLC spots to the mass detector. The mass detector was operated in positive mode (ESI+) and the fragmentor voltage was 60 V. Detection was performed in full scan mode.

### Stability study

The stability of five replicate samples of HHMA at concentrations of 100 and 10 ng/mL, at ambient temperature (25°C) and 0°C, in both perfusion medium and water was examined for 24 hours. In the case of perfusion medium, the stability was checked at 0, 5, 15, 30, 60, 120 and 180 min, and in the case of water, samples were taken at 0, 5, 15, 30, 60, 120, 180, 240 min and 24 hours. These samples were analyzed by the HPLC/FL for the determination of HHMA residual in corresponding medium.

The impact of perfusion medium components on HHMA stability was examined separately on 5 replicate samples (at a concentration appeared in perfusion medium) of the 100 ng/mL aqueous solution of HHMA. Samples were analyzed immediately after dissolving the component by the HPLC/FL and were compared with the 100 ng/mL freshly prepared samples of HHMA in water. The influence of pH (3–10) and different concentration (up to saturated concentration) of ethylenediaminetetraacetic acid (EDTA) on the stability of HHMA in perfusion medium was also investigated.

Moreover, the stability of HHMA in water in the presence of calcium chloride (CaCl_2_) (concentration 0–150 g/L) was studied. Samples were analyzed by HPLC/UV immediately after the changes were made and the results were compared with the results obtained for freshly prepared samples of HHMA in corresponding medium. This part was done on 5 replicate samples of 1 μg/mL solution of HHMA.

The effect of CaCl_2_ (150 g/L) on the stability of HHMA aqueous solution (1 μg/mL) was also examined by HPTLC system on 3 replicate samples.

In another part of the present study, those components which were supposed to be responsible for instability of HHMA in perfusion medium were also examined, for their impact on the stability of dopamine (1 μg/mL aqueous solutions).

### Statistics

The t-independent test was used for determination of differences between means of groups (P < 0.05).

## Results and discussion

**Figure 3:**
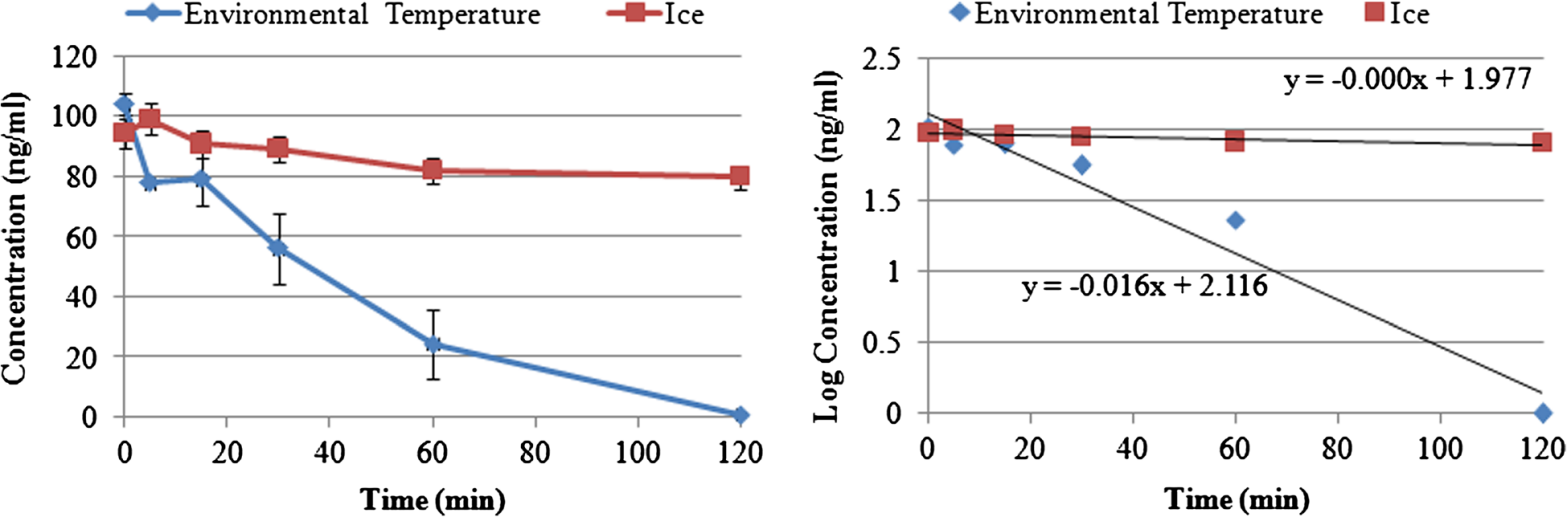
**The degradation rate of 100 ng**/**mL solution of HHMA in perfusion medium at ambient temperature and 0°C.** Points shown as mean ± SD, n=5

The 100 and 10 ng/mL solutions of HHMA in water were stable at ambient temperature and 0°C for 24 hours. No significant differences between area under the curve (AUC) of peak of freshly prepared samples and tests were observed (n = 5, P > 0.05), whereas the 100 and 10 ng/mL solutions of HHMA in perfusion medium were unstable. After 2 hours, about 100% of the 100 ng/mL solution of HHMA was degraded at ambient temperature (K_degradation_ = 0.037 min^-1^ (0.030-0.047) at 25°C) and a new small peak appeared in the HPLC/FL chromatogram. Reduction of the temperature to 0°C could reduce the rate of degradation, so that after 2 hours, only about 20% of the 100 ng/mL solution of HHMA was degraded (K_degradation_ = 0.002 min^-1^ (0.0006-0.003) at 0°C) (Figure 3). In the case of 10 ng/mL solution of HHMA in perfusion medium, the similar pattern was observed.

**Figure 4:**
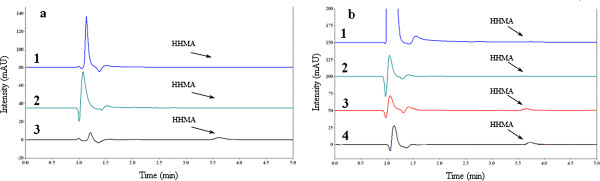
**The effect of pH on degradation of HHMA (1 μg/mL) in water (a) and perfusion medium (b) at ambient temperature.** a1: at pH=9; a2: changing the pH of alkaline sample to 3; a3: at pH=3; b1: at pH=9; b2: changing the pH of alkaline sample to 3; b3: at pH=3; b4: at pH=7.3.

While the reduction of pH to 3 could stop the degradation reaction, increasing the pH could accelerate degradation rate, in such a way that the corresponding peak to HHMA in HPLC/FL or HPLC/UV chromatograms disappeared rapidly after pH was reached to 9. The HHMA peak disappearance was permanent and by reducing the pH of the alkaline samples to 3, the HHMA peak did not appear again (Figure 4).

Our results were in accordance with Gouarne et al. report [14]. They reported that the degradation rate of catecholamins is in direct proportion with pH of medium. The hydroxyl groups in the catecholamine structure deprotonate easier at pHs higher than 3 and form the corresponding quinone structure readily. At high pHs, quinine structures are susceptible to oxidation, cyclization and polymerization [10,11]. It should be mentioned that the pH of aqueous solutions of HHMA was about 3 and perhaps due to this reason, HHMA has been stable in these aqueous solutions.

Adding EDTA to the HHMA solution in perfusion medium, even at a saturated concentration could only reduce the rate of degradation about 30%. It has been reported that MgCl_2_ and CaCl_2_ enhances the oxidation of ascorbate [15]. EDTA, due to its chelating property, can reduce the rate of oxidation reaction by sequestering the Ca^2+^ and Mg^2+^, but this chelator was not effective in inhibition of HHMA peak reduction.

In order to further investigate this phenomenon, the components of perfusion medium (at their concentrations in perfusion medium) were added to HHMA aqueous solution separately. Adding MgSO_4_, NaCl, KCl, KH_2_PO_4_ or glucose did not affect the AUC of HHMA peak even after 2 hours. These results indicated that Mg^2+^, which is a crucial cation in Catechol-O-methyltransferase (COMT, an enzymes that play a role in catecholamine degradation) activity [16] and can bind to hydroxyl ring of catecholamine at pHs higher than 7 [17], has no effect on HHMA instability. However, CaCl_2_ and NaHCO_3_ were the components which caused HHMA concentration to reduce. In case of NaHCO_3_, the reduction of HHMA concentration was expected due to its ability in increasing the pH of solutions. However, the observed rate for concentration reduction of HHMA, was slower than the observed rate for HHMA in perfusion medium. In addition to NaHCO_3_, CaCl_2_ could cause reduction in concentration of HHMA in aqueous solution. When the concentration of CaCl_2_ increased from 0.3 to 150 g/L, the AUC of HHMA peak reduced faster. Moreover, increasing the shaking time from 5 to 60 seconds reduced the AUC of HHMA peak, significantly.

**Figure 5:**
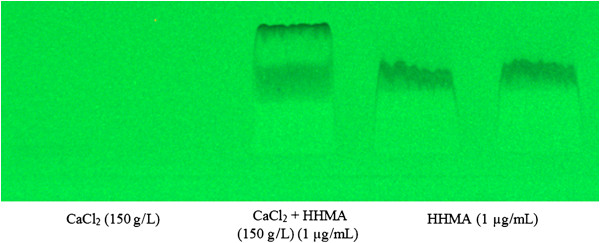
**The HPTLC chromatogram of HHMA (1 μg/mL) in water and in the presence of CaCl**_**2 **_**(150 g/L).**

**Figure 6:**
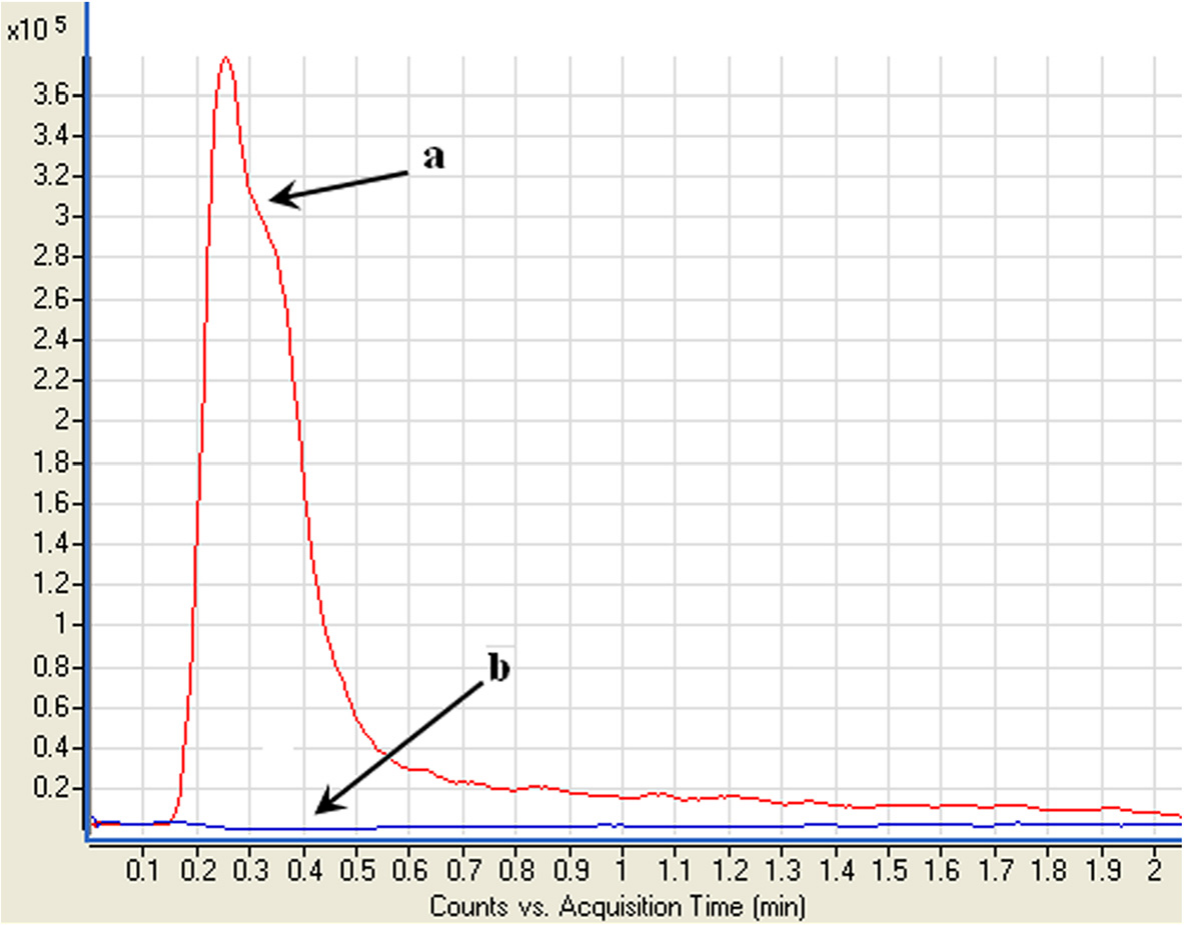
**Extracted ion chromatogram at (*m/z*182) of HHMA (1 μg/mL) in the absence (a) and in the presence (b) of CaCl**_**2 **_**(150 g/L).**

The HPTLC analysis of HHMA aqueous solution in the presence of CaCl_2_ showed that a new spot appeared in the chromatogram after adding CaCl_2_ (150 g/L) with the Rf = 0.25. This spot did not exist in the chromatograms of HHMA or CaCl_2_ aqueous solutions alone. Mass spectroscopic analysis of this spot on a triple quadruple mass detector revealed that its molecular weight is different from that of HHMA. Moreover, the UV spectrum of this spot was different from the UV spectrum of HHMA aqueous solution (Figures 5 and 6). The same results were obtained in all replication. These results indicated that HHMA has changed to other compounds in the presence of CaCl_2_. There are no reports in the literature about a direct role of CaCl_2_ in oxidation of catecolamines. It seems that Cl^-^ has no impact on stability of HHMA, because adding NaCl could not reduce the AUC of HHMA peak over 2 hours. It could be speculated that calcium cation (Ca^2+^), due to free d-orbital in its electronic configuration, can act as a catalyst or something like that in this phenomenon, but magnesium cation (Mg^2+^), due to the lack of this free d-orbital, cannot play such role.

It has been reported that HHMA can be oxidized to related ortho quinone compounds that react with nucleophilic groups of macromolecules or form neurotoxins [8,18]. Moreover, HHMA can change to carbene intermediate and covalently bind to heme iron of CYP2D6 in the biological systems [18,19]. Beside HHMA, dopamine, which is structurally the most similar compound to HHMA, is oxidized in the presence of MnO_2_ and NaIO_4_ to the reactive ortho quinone compounds which covalently bind to cysteinyl group of proteins and impair their functions. Moreover, reactive ortho quinone compounds are responsible for neurodegenerative process of dopaminergic system. When the oxidizing agents were added to the aqueous solutions of dopamine, the absorption intensity at λ = 280 nm decreased and two new peaks appeared at λ = 300 and 475 nm in the UV spectrum, which were associated with AC structure [10,11]. The similar pattern changes were reported in the UV spectrum of HHMA when oxidized electrochemicaly [8]. In our study, such a phenomenon was observed when the CaCl_2_ was added to aqueous solution of dopamine. In the UV spectrum when CaCl_2_ (150 g/L) was added to 1 μg/mL dopamine aqueous solution, the intensity of the absorption at λ = 276 nm decreased, and after 10 min a new peak at λ = 294 and a small peak at about 455 nm appeared.

In light of the above observations, it could be speculated that HHMA, in the presence of CaCl_2_, is oxidized to its corresponding aminochrome like structures. Therefore, in study of MDMA effect on isolated organs, the perfusion medium should not contain CaCl_2_ or Ca^2+^. However, calcium cation has a crucial role in cell biological activity, vesicle movement and exocytosis [20]. The total vesicular concentration of Ca^2+^ is about 1.6 g/L and the main internal source of this cation is endoplasmic reticulum. In addition to, CYP450 isoenzymes are located and activated in endoplasmic reticulum. Therefore, the role of this cation in generating reactive ortho quinonic compounds and neurotoxicity associated with MDMA could be speculated.

## Conclusion

Serotonin neuron damage which is related to the MDMA metabolites is one of crucial aspect of MDMA side effects. Different reports have been published about this effect, some of them suggesting HHMA as the neurotoxic metabolite of MDMA, while other reports suggest MDA as the neurotoxin. One of the possible reasons for this controversy can be due to failure in HHMA determination as a free form in these studies.

The results of our study indicated that Ca^2+^ besides sodium bicarbonate can be responsible components in HHMA concentration reduction in perfusion medium. In order to obtain reliable results in these cases, the perfusion medium should not contain Ca^2+^ and the pH of medium is better to be adjusted at acidic range. Moreover, Ca^2+^can play a role in dopamine conversion to AC, which has a distinctive role in neurodegenerative process of dopaminergic system. In summary, it would be possible that Ca^2+^ accelerates HHMA degradation or plays a role in producing neurodegenerative diseases associated with dopamine or MDMA neurotoxicity.

## Competing interests

The authors declare that they have no competing interests.

## Authors’ contributions

M-RR, YHA and BJ conceived the study. AF synthesized the required chemicals which were not available from chemical companies, BJ, YHA, SA, VHZA and FK performed the experimental work. All authors were involved in data analysis and interpretation. BJ, M-RR and FK drafted the manuscript. All authors read and approved the final version.
